# Injection of Vaseline under Penis Skin for the Purpose of Penis Augmentation

**DOI:** 10.1155/2012/510612

**Published:** 2012-11-19

**Authors:** Tolga Karakan, Erim Ersoy, Metin Hasçiçek, Berat Cem Özgür, Serkan Özcan, Arif Aydın

**Affiliations:** Urology Clinic, Ankara Training and Research Hospital, Ministry of Health, 06260 Ankara, Turkey

## Abstract

Penile foreign body injection is an uncommon entity produced by penile paraffin, mineral oil, and vaseline injections for the purpose of penile enlargement. Generally, penile subcutaneous and glandular injections for penile augmentation are performed by a nonmedical person, under unacceptable conditions. It will be an aim to share our experiences about penile vaseline injection.

## 1. Introduction

Bigger sexual organ was the symbol of power in many cultures. Injection of foreign substances into penis in order to make the sexual organ appears bigger is reported especially in Asian and Eastern European countries [1]. Paraffin, Vaseline and mineral oil, and so forth are generally used as foreign substances [2]. In some of the Asian countries, placing small chagan balls under penis skin in order to improve sexual pleasure of the partner can be seen especially among men with lower socioeconomic level. Years after the injection, granulomatous skin reaction referred to as vaselinome or paraffinom may occur and adverse effects such as infection, ulceration, fistula, local migration, and cavernous invasion may also occur [3]. In diagnosing foreign substance reaction, venereal diseases and squamous-cell carcinoma should be noted [4]. Injection of foreign substances may be a predisposing factor for Fournier gangrene, even very rare [5].

 The most important step during the cure is to completely dissect and pull out the foreign substance [6]. Then, if primary enclosure is possible, it should be done or it should be repaired by scrotal flap or full layer skin grafts. Antibiotic therapy is only important in controlling secondary infections. Some authors stated that formation of sclerosant lipogranuloma can be cured best by corticosteroids [7]. 

## 2. Case Presentation

**Figure 1:**
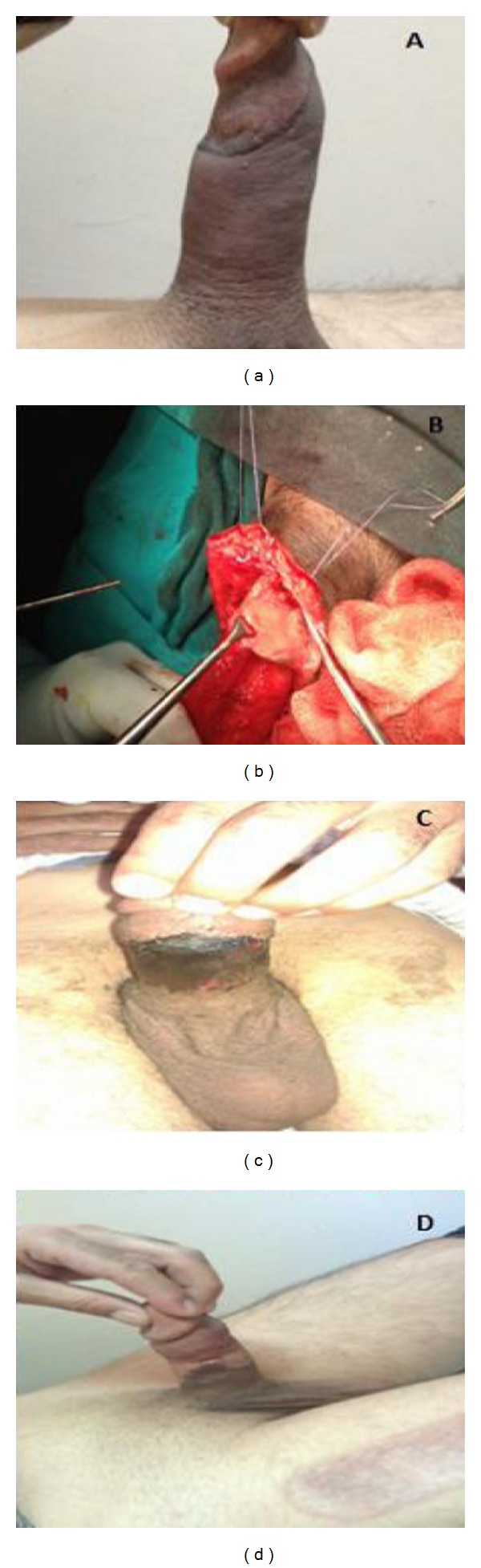
(A) Appearance of penis one week after the injection. (B) Dissection of vaseline (C). Postoperative seventh day. (D) Split thickness skin graft.

A 42-year-old male patient had applied to our clinic with a claim of pain in his penis. It is understood according to patient's history that he had a multiple heated Vaseline injection done under penis skin one month ago for augmenting his penis. In patient's physical examination, it was palpated that there were soft-formed fixed and widespread mass lesions under penis skin. No deformation was found on penis skin surface (Figure 1). Patient stated that he had normal erection but he felt pain during sexual relation. In preoperative examinations done on the patient, no biochemical or hematological disorder was detected. 

 In patient's superficial tissue ultrasonography, a thickening nearly 10 mm and obvious eco increase at extratunical level on penis root and penile shaft proximal section's dorsal side and tissue field with similar appearance on penile shaft distal section's ventral side were reported. No pathology was detected on both corpus cavernosum and corpus spongiosum. 

It was decided to make an operation on the patient in order to pull out the foreign substance. Degloving was done to penis with circumcision incision after intraoperative urethral catheter was inserted and it was seen that tunica albugiane was intact. However, vaseline was spreaded among the subcutaneous layers to the highest degree and caused cohesion and granulomatous reaction. Vaseline was pulled out by being separated from tissues through dissection and necrotic fields which were excited. Layers were enclosed with anatomic plan after all foreign substances were pulled out and penis was bandaged up with an elastic bandage. On second postoperative day, the patient whose bandage was opened had been discharged from hospital as his wound appearance was seen better, with antibiotics and anti-inflammatory medication therapy. It was seen that penis skin was going under necrosis on the 7th postoperative day during patient followup and patient's consultation is done at plastic surgery clinic. Patient again hospitalized by plastic surgery clinic, was operated again as circular necrosis was reformed on penis skin during patient follow-up process, and defect repair was done with split thickness skin graft prepared from right-anterior femoral skin.

## 3. Discussion

Reasons for penis augmentation may be listed as erectile disfunction, improving partner's sexual pleasure, improving self-confidence feeling, and braveness when the literature is searched [8]. As it is seen in our incident, it is very important to make surgical excision in the early period and pulling foreign substance out in terms of preventing serious complications likely to occur on long term for such cases. The possibility of skin necrosis formation and requirement for additional cures after excision depending on penetration of foreign substance and degree of the dissection done should be noted.
